# Gender Differences in the Association Between Screen Time and Depression

**DOI:** 10.1016/j.focus.2023.100176

**Published:** 2023-12-20

**Authors:** Lauren E. Kleidermacher, Mark Olfson

**Affiliations:** 1 Columbia University Vagelos College of Physicians & Surgeons, New York, New York; 2 Mailman School of Public Health, Columbia University, New York, New York; 3 New York State Psychiatric Institute, New York, New York

**Keywords:** Gender, screen time, depression, computer, TV

## Abstract

**Introduction:**

Previous research has shown that screen time is associated with depression, especially in children. Some evidence further suggests that the association may be stronger in women than in men, although findings are inconclusive. This cross-sectional study examines the association between screen time and depression in representative U.S. adults, stratified by gender.

**Methods:**

This study used data from the 2015–2016 National Health and Nutrition Examination Survey; analysis was conducted in 2023. Screen time was partitioned into 3 categories—0–2 hours, 3–4 hours, and >4 hours—and included TV and computer time. Depression was defined as a Patient Health Questionnaire score ≥10. TV time and computer time were also analyzed as separate exposures. A multivariable logistic regression model examined the association between screen time and depression.

**Results:**

Results showed that there was a significant interaction between gender and screen time. An association between the highest screen time exposure group and depression was observed for women (>4 hours per day: OR=3.09; 95% CI=1.68, 5.70). The type of screen time affected the relationship, with TV showing a stronger association than computer time. There were no significant associations in men across all exposure groups.

**Conclusions:**

Further research is needed to determine whether higher levels of screen time, especially TV, may be a depression risk marker for women but not men.

## HIGHLIGHT

• Screen time has been found to be associated with depression in previous studies.
• Longer screen time was associated with depression for women but not for men.
• Screen time levels may be a useful indicator of depression in certain populations.

## Graphical Abstract

**Figure:**
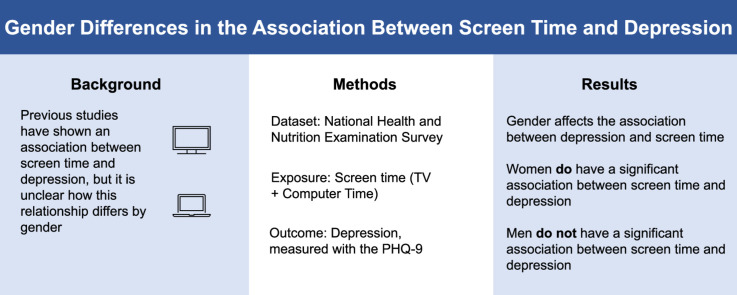

## INTRODUCTION

Several reports have shown a positive association between screen time and depression.^1^, ^2^, ^3^ A meta-analysis further reported that this association, although similar in both genders, was significant in women (OR=1.18; 95% CI=1.03, 1.35) but not in men (OR=0.96; 95% CI=0.63, 1.47).^2^^,^^3^ However, this research has primarily relied on convenience samples and younger populations; therefore, uncertainty exists concerning the generalizability of the association to adults and whether screen time is more strongly connected to depression in women than in men. Furthermore, because research on whether sedentary behavior can cause weight gain and whether weight gain can cause depression remains elusive, there is also uncertainty about whether BMI mediates the relationship between screen time and depression.^4^, ^5^, ^6^, ^7^

Within a nationally representative population of U.S. adults, we examined whether the association between screen time and depression differs by gender or type of screen time and evaluated whether BMI, which is related to physical inactivity and depression, partially mediates this association.^8^^,^^9^

## METHODS

### Study Population

Data were analyzed from the 2015–2016 National Health and Nutrition Examination Survey (NHANES), a U.S. nationally representative cross-sectional survey.^10^ Recruitment for the 2015–2016 NHANES (N=15,327) is detailed elsewhere.^10^ In short, health interviews were conducted by trained personnel at participants' homes, whereas health measurements were performed in mobile centers.^10^ Data were acquired about participants' sociodemographic characteristics, mental health, and self-reported screen time. The response rate was 61.3%, and those aged ≥60 years, African Americans, Asians, and Hispanics were oversampled.^10^ The New York State Psychiatric IRB determined that this analysis was exempt from human subject review.

### Measures

The pirmary outcome, depression, was assessed with the Patient Health Questionnaire (PHQ-9), a self-report measure of DSM major depressive disorder symptoms in the last 2 weeks from 0 (not at all) to 3 (nearly every day).^11^ At a cut score of 10, the PHQ-9 has a sensitivity and specificity of 88% for major depressive disorder.^12^

Gender was the stratification variable, and screen time, queried as the total hours participants reported spending on their computer or watching TV, was the primary exposure. Screen time was classified as 0–2 hours, 3–4 hours, and >4 hours. Secondary exposures included computer time and TV time as separate exposures (0–2 hours, 3–4 hours, and >4 hours).

Covariates included age (20–35, 36–50, 51–65, ≥66 years), education (less than high school/GED, high school/GED or equivalent, college graduate or above), race/ethnicity (Mexican American, other Hispanic, non-Hispanic White, non-Hispanic Black, other), and poverty level (above or below the U.S. poverty threshold).^13^

### Statistical Analysis

Background characteristics of male and female respondents by screen time exposure status were first examined. Multivariate logistic regression models were fit to evaluate the strength of associations of each level of screen time exposure, with 0–2 hours as the reference group and depression as the outcome, adjusted for age, education, race/ethnicity, and poverty level. Effect modification by gender was tested using contrasts of marginal linear predictions. All analysis was stratified by gender. BMI was later added to the model to test for mediation (4 groups: underweight [<18.5 kg/m**^2^**], normal weight [18.5–24.9 kg/m**^2^**], overweight [25–29.9 kg/m**^2^**], and obese [>30 kg/m**^2^**]).

Participants with 1 missing value from the PHQ-9 were included with a mean imputation calculation, whereas those with >1 missing answer were excluded. Participants with missing computer and TV screen time were also excluded. To retain power, participants with missing demographic information were analyzed as a separate missing category. Outliers were examined with box plots and histograms. Analyses were performed with Stata, Version 17, employing SUDAAN to accommodate the complex survey design and weighted sampling in 2023 (Stata, version 17).

## RESULTS

Among participants with PHQ-9 responses (*n*=5,158), 13 had incomplete exposure data, leaving 5,145 in the final analysis. Overall, 8.1% of participants had depression, including 9.7% of women and 6.6% of men (data not shown in tables). Women aged ≥51 years were more likely to watch >4 hours of screen time than younger women (Table 1). Women with a high school education/GED had higher screen time exposure than those in other education groups. Hispanic women had lower screen time than non-Hispanic White and non-Hispanic Black women. Women who were obese were more likely to watch >4 hours per day of screen time than those with normal weight. No notable screen time differences were observed between income groups for women. Screen time for men followed a similar pattern, although men were more likely to have higher values of screen time across all age groups than those aged 36–50 years, and the difference between obese and normal-weight individuals was not as large.

**Table 1:** Baseline Characteristics in Women and Men, Overall and by Screen Time Groups

	Women (*n*=2,692)			Men (*n*=2,516)		
Characteristics	0–2 h/day (*n*=1,028)	3–4 h/day *(n*=750)	>4 h/day (*n*=851)	0–2 h/day (*n*=914)	3–4 h/day (*n*=746)	>4 h/day (*n*=856)
Age, years						
20–35	314 (41.2)	239 (32.0)	232 (26.8)	291 (40.1)	206 (27.8)	276 (32.2)
36–50	322 (51.8)	165 (25.4)	153 (22.8)	259 (45.7)	169 (31.1)	129 (23.1)
51–65	251 (32.3)	174 (27.5)	220 (40.2)	223 (34.7)	199 (30.3)	216 (35.0)
≥66	141 (18.0)	172 (32.1)	246 (50.0)	141 (18.6)	172 (36.4)	235 (45.0)
Education						
Less than high school/GED	285 (45.4)	131 (23.3)	133 (31.3)	268 (44.3)	164 (27.1)	149 (28.6)
High school/GED or equivalent	443 (31.6)	399 (28.9)	493 (39.5)	406 (34.4)	341 (29.0)	470 (36.6)
College graduate or above	265 (43.7)	186 (31.4)	175 (25.0)	209 (37.4)	205 (35.3)	176 (27.3)
Race						
Mexican American	265 (54.0)	136 (28.3)	92 (17.7)	210 (48.7)	126 (26.9)	102 (24.5)
Other Hispanic	172 (47.2)	112 (30.7)	85 (22.2)	131 (51.1)	82 (24.9)	76 (24.0)
Non-Hispanic White	279 (35.1)	246 (29.4)	314 (35.5)	276 (34.7)	271 (31.9)	327 (33.4)
Non-Hispanic Black	168 (30.2)	159 (28.3)	242 (41.6)	141 (28.3)	155 (28.9)	236 (42.8)
Other race	144 (35.8)	97 (28.6)	118 (35.7)	156 (36.8)	112 (32.3)	115 (30.9)
Poverty						
Below the poverty threshold	238 (37.6)	144 (26.7)	185 (35.7)	189 (35.9)	118 (24.5)	177 (39.6)
Above the poverty threshold	683 (37.2)	522 (29.0)	590 (33.8)	636 (36.6)	559 (31.7)	594 (31.7)
BMI (kg/m^2^)						
Underweight (<18.5)	19 (33.5)	12 (30.5)	17 (36.1)	7 (18.6)	10 (35.4)	14 (46.0)
Normal weight (18.5–24.9)	237 (40.9)	170 (32.3)	170 (26.8)	204 (42.0)	143 (27.0)	167 (31.1)
Overweight (25–29.9)	300 (41.4)	193 (28.8)	197 (29.8)	348 (38.8)	289 (33.1)	279 (28.1)
Obese (>30)	462 (32.2)	371 (28.0)	455 (39.8)	351 (33.0)	299 (30.3)	382 (36.7)

*Note:* Data are presented as *n* (%). Characteristic groups are the following order: age, education, race, poverty, and BMI (kg/m^2^). The following characteristics had missing values, where participants either refused to answer or did not answer: education, 119 women and 128 men; poverty, 267 women and 243 men; and BMI, 26 women and 23 men. Data are from the 2015–2016 NHANES data set; % calculations include sample weights.

h, hours; NHANES, National Health and Nutrition Examination Survey.

**Figure 1:**
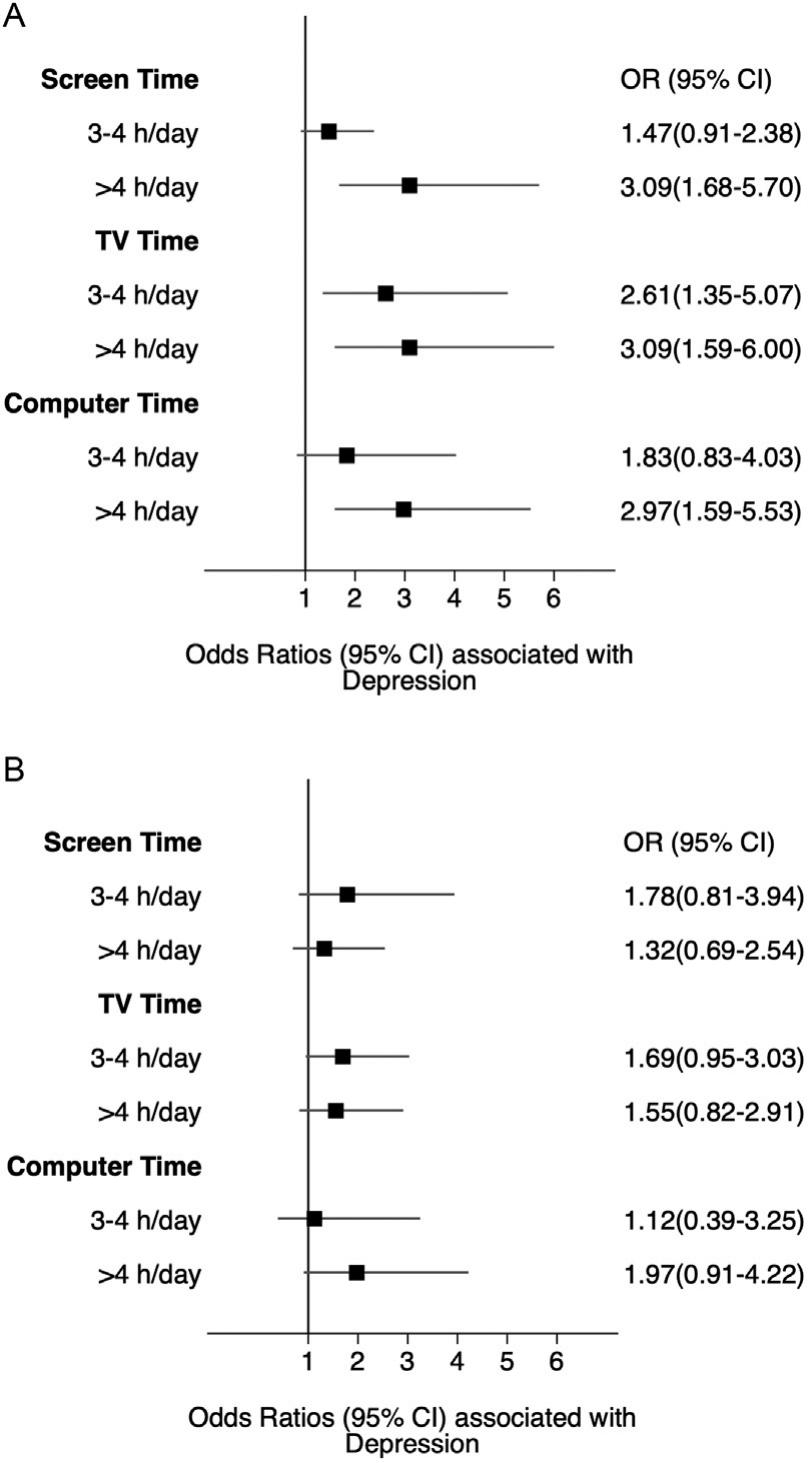
Association between screen time and depression, stratified by gender. (A) Women. (B) Men. Cross-sectional associations of self-reported screen time with depression in (A) women and (B) men are shown. Models were adjusted for age, education, poverty level, and race. Each square symbolizes an OR; 95% CIs are plotted as error bars on each square. Data are from the 2015–2016 NHANES data set. Screen time is defined as the combination of TV and computer time. Reference groups are 0–2 hours of screen time. NHANES, National Health and Nutrition Examination Survey.

The adjusted odds of depression were significantly higher for women who reported screen time of >4 hours per day than for those who reported ≤2 hours per day (OR=3.09; 95% CI=1.68, 5.70). (Figure 1A). In addition, the adjusted odds of depression for women varied by screen time type. TV exhibited a graded response (3–4 hours per day, OR=2.61 [95% CI=1.35, 5.07] and >4 hours per day, OR=3.09 [95% CI=1.59, 6.00]), whereas there was no association between computer time and depression until >4 hours was reached (OR=2.97; 95% CI=1.59, 5.53).

In contrast, screen time, computer time, or TV time for men were not significantly associated with depression (Figure 1B). There was an interaction between gender and the association between screen time and depression (*p*<0.01).

When BMI was added to the model, the adjusted odds of depression for women were attenuated to 2.87 (95% CI=1.57, 5.24) for >4 hours per day of screen time (<3 hours per day, reference). This attenuation was also seen between TV time and depression for women (3–4 hours/day: OR=2.55 [95% CI=1.33, 4.85]; >4 hours per day: OR=2.85 [9%% CI=1.45, 5.61]) and computer time (>4 hours per day: OR=2.85 [95% CI=1.59, 5.10]).

## DISCUSSION

In this nationally representative survey, the association between screen time and self-reported depression was stronger for women than for men, although CIs were wide. Among women, there was also variation across screen types in the association with depression. Specifically, TV showed a graded response for women, whereas computer time did not; there was only an association when greater computer times were reached. Finally, BMI attenuated the association between screen exposure and depression, raising the possibility that it contributes to the connection between screen time and depression.

High screen time may be an antecedent or consequence of depression; longitudinal research is needed to distinguish the relative importance and gender differences in these 2 causal pathways. In support of depression increasing screen time differentially in women, prospective studies demonstrate that depression leads to greater weight gain in women than in men, and weight gain is closely connected with sedentary behavior such as TV viewing.^14^^,^^15^ In support of screen time causing depression, a recent meta-analysis of prospective studies found that sedentary behavior, especially passive behavior such as TV watching, is associated with an increased risk of depression.^16^ Longitudinal studies have not yet examined whether these risks vary by gender.

### Limitations

Several limitations constrained this study. First, temporality could not be determined in this cross-sectional survey. In addition, self-reported screen time might have been over- or under-reported.^17^ There also may have been residual confounding by other psychiatric conditions or other factors. Although the results reflect the most recent NHANES data to probe screen time, these 2015–2016 findings may not reflect contemporary associations between screen time and depression. In addition, the data set did not specify whether computer time was recreational or occupational. Finally, the 61.3% response rate may compromise generalizability.

## CONCLUSIONS

Screen time is associated with depression for women but not for men in the U.S. These findings highlight the potential of screen time assessments to identify women at increased risk of depression.

## CRediT authorship contribution statement

**Lauren E. Kleidermacher:** Conceptualization, Data curation, Formal analysis, Methodology, Software, Validation, Visualization, Writing – original draft. **Mark Olfson:** Methodology, Writing – review & editing.

## Appendix Supplementary materials

Image, application 1
