# Supplementary material for: Gender Differences in the Association Between Screen Time and Depression
Source: AJPM Focus. 2023 Dec 20;3(2):100176. doi: 10.1016/j.focus.2023.100176 (PMC10831171; doi:10.1016/j.focus.2023.100176)
Supplement: Supplementary file 1 [file mmc1.docx]

STROBE Statement—checklist of items that should be included in reports of observational studies

|  | Item No. | Recommendation | Page  No. | Relevant text from manuscript |
| --- | --- | --- | --- | --- |
| **Title and abstract** | 1 | (*a*) Indicate the study’s design with a commonly used term in the title or the abstract | 2 | This cross-sectional study |
|  |  | (*b*) Provide in the abstract an informative and balanced summary of what was done and what was found | 2 | Methods and Results section |
| Introduction | | | |  |
| Background/rationale | 2 | Explain the scientific background and rationale for the investigation being reported | 3 | A meta-analysis further reported that the association between screen time and depression, while similar in both genders, was significant in women (OR=1.18, 1.03-1.35), but not in men (OR=0.96, 0.63-1.47) |
| Objectives | 3 | State specific objectives, including any prespecified hypotheses | 3 | This study examines among nationally representative US adults whether the strength of the association between screen time and depression differs by gender or type of screen time activity, while also considering whether body mass index (BMI), which is related to physical inactivity and depression, partially mediates this association |
| Methods | | | |  |
| Study design | 4 | Present key elements of study design early in the paper |  |  |
| Setting | 5 | Describe the setting, locations, and relevant dates, including periods of recruitment, exposure, follow-up, and data collection | 4-5 | Population section in methods |
| Participants | 6 | *Cross-sectional study*—Give the eligibility criteria, and the sources and methods of selection of participants | 4-5 | Population section in methods, ex: The 2015-2016 NHANES is a cross-sectional study that sampled 15,327 participants of all ages. |
| Variables | 7 | Clearly define all outcomes, exposures, predictors, potential confounders, and effect modifiers. Give diagnostic criteria, if applicable | 4-5 | Under “Measures” Section |
| Data sources/ measurement | 8* | For each variable of interest, give sources of data and details of methods of assessment (measurement). Describe comparability of assessment methods if there is more than one group | 4-5 | Under outcome and exposure subheadings |
| Bias | 9 | Describe any efforts to address potential sources of bias | 4-5 | The response rate was 61.3%, and those 60 and over, African Americans, Asians, and Hispanics, were oversampled to allow for greater precision of survey estimates of these groups. |
| Study size | 10 | Explain how the study size was arrived at | 5-6 | Participants with one missing value from the PHQ-9 were included with a mean imputation calculation, while those with more than one missing answer were excluded. Participants with missing computer and television screen time were also excluded. Participants with missing demographic information were included and analyzed as a separate “missing” category. |

| Quantitative variables | 11 | Explain how quantitative variables were handled in the analyses. If applicable, describe which groupings were chosen and why | 4-5 | Included in “Measures” section, specifically in the outcome, exposure and stratification, and background characteristics subheadings. |
| --- | --- | --- | --- | --- |
| Statistical methods | 12 | (*a*) Describe all statistical methods, including those used to control for confounding | 5 | Explained in the statistical analysis subheading |
|  |  | (*b*) Describe any methods used to examine subgroups and interactions |  | Effect modification with gender was tested using contrasts of marginal linear predictions |
|  |  | (*c*) Explain how missing data were addressed | 5-6 | Participants with one missing value from the PHQ-9 were included with a mean imputation calculation, while those with more than one missing answer were excluded. Participants with missing computer and television screen time were also excluded. Participants with missing demographic information were included and analyzed as a separate “missing” category. |
|  |  | *Cross-sectional study*—If applicable, describe analytical methods taking account of sampling strategy | 6 | Analyses were done using Stata version 17 employing SUDAAN to accommodate the complex survey design and weighted sampling in 2023 (Stata, version 17). |
|  |  | (*e*) Describe any sensitivity analyses |  | N/A |
| Results | | | | |
| Participants | 13* | (a) Report numbers of individuals at each stage of study—eg numbers potentially eligible, examined for eligibility, confirmed eligible, included in the study, completing follow-up, and analysed | 7 | A total of 5,145 participants were included in the final analysis. |
|  |  | (b) Give reasons for non-participation at each stage | 5-6 | Missing variables previously explained in methods section. |
|  |  | (c) Consider use of a flow diagram |  | N/A |
| Descriptive data | 14* | (a) Give characteristics of study participants (eg demographic, clinical, social) and information on exposures and potential confounders | 7 | Results, paragraph 1 |
|  |  | (b) Indicate number of participants with missing data for each variable of interest | 7 | Of the total population, 5,158 participants responded to the PHQ-9, and of these participants, 13 people incomplete exposure data. A total of 5,145 participants were included in the final analysis.  Tale 1 footnote. |
|  |  | *Cross-sectional study—*Report numbers of outcome events or summary measures | 7,11 | Overall, 8.14% of respondents had depression including 9.7% of women and 6.6% of men Table 1 |
| Main results | 16 | (*a*) Give unadjusted estimates and, if applicable, confounder-adjusted estimates and their precision (eg, 95% confidence interval). Make clear which confounders were adjusted for and why they were included | 7,8 | The adjusted odds of depression were significantly higher for women who reported >4 than ≤2 hours/day of screen time (OR, 95%CI=3.09, 1.68-5.70). (Figure 1A). In addition, the adjusted odds of depression for women varied by screen time type. TV exhibited a graded response: 3-4 hours/day, 2.61(1.35-5.07); >4 hours/day 3.09(1.59-6.00), while there was no association between computer time and depression until >4 hours was reached, 2.97(1.59-5.53).  Figure 1 |
|  |  | (*b*) Report category boundaries when continuous variables were categorized |  | Explained in (a) |
|  |  | (*c*) If relevant, consider translating estimates of relative risk into absolute risk for a meaningful time period |  | N/A |

| Other analyses | 17 | Report other analyses done—eg analyses of subgroups and interactions, and sensitivity analyses | 7 | There was an interaction between gender and the association between screen time and depression (p<0.01). |
| --- | --- | --- | --- | --- |
| Discussion | | | | |
| Key results | 18 | Summarise key results with reference to study objectives | 8 | In this nationally representative cross-sectional study, the association between screen time and self-reported depression was significantly stronger for women than men. Among women, there was also variation across screen types in the association with depression. Specifically, TV showed a graded response for women, while computer time did not; there was no association until greater exposure were reached. Finally, BMI attenuated the association between each screen exposure and depression, raising the possibility that it contributes to the connection between screen time and depression. |
| Limitations | 19 | Discuss limitations of the study, taking into account sources of potential bias or imprecision. Discuss both direction and magnitude of any potential bias | 8,9 | Several limitations constrained this study. First, as this is a cross-sectional study, temporality could not be determined. Second, self-reported screen time might have been over- or under-reported^13^. Third, there may have been residual confounding (such as anxiety or other psychiatric conditions). Finally, the 61.3% response rate may compromise generalizability. |
| Interpretation | 20 | Give a cautious overall interpretation of results considering objectives, limitations, multiplicity of analyses, results from similar studies, and other relevant evidence | 8,9 | High screen time may be an antecedent or consequence of depression; longitudinal research is needed to distinguish the relative importance of these two causal pathways and why they appear to function differently in women and men. In support of depression increasing screen time differentially in women, prospective studies demonstrate depression leads to greater weight gain in women than men, and weight gain is closely connected with sedentary behavior such as television viewing.^10,11^ In contrast, in support of screen time causing depression, a recent meta-analysis of prospective studies found that sedentary behavior, especially passive behavior such as television watching, is associated with an increased risk of depression.^12^ Longitudinal studies have not yet examined whether these risks vary by gender. |
| Generalisability | 21 | Discuss the generalisability (external validity) of the study results | 8 | In this nationally representative cross-sectional study |
| Other information | |  | | |
| Funding | 22 | Give the source of funding and the role of the funders for the present study and, if applicable, for the original study on which the present article is based | 10 | This study was supported with funds from the Vagelos College of Physicians and Surgeons by the Department of Medical Education, which did not have a role in study design, data analysis, writing, or paper submission. |

*Give information separately for cases and controls in case-control studies and, if applicable, for exposed and unexposed groups in cohort and cross-sectional studies.

**Note:** An Explanation and Elaboration article discusses each checklist item and gives methodological background and published examples of transparent reporting. The STROBE checklist is best used in conjunction with this article (freely available on the Web sites of PLoS Medicine at http://www.plosmedicine.org/, Annals of Internal Medicine at http://www.annals.org/, and Epidemiology at http://www.epidem.com/). Information on the STROBE Initiative is available at www.strobe-statement.org.
